# Mental capacity in Colombia: a comparison with the UK

**DOI:** 10.1192/bji.2017.17

**Published:** 2018-11

**Authors:** Juan P. Borda, Ricardo Tamayo, Gareth Owen

**Affiliations:** 1 Corporación Universitaria Empresarial Alexander von Humboldt, Faculty of Medicine, Armenia, Colombia. Email: juanpablobordab@gmail.com; 2 Consultant Psychiatrist, Instituto Nacional de Medicina Legal y Ciencias Forenses, Bogotá, Colombia; 3 Clinical Senior Lecturer in Mental Health, Ethics and Law, Wellcome Trust Senior Research Fellow, Institute of Psychiatry, Psychology and Neuroscience, King's College London

## Abstract

Several international proclamations in the last decades have advocated for the dignity and autonomy of persons with mental disorders. Few discussions have been generated regarding the implication of this transition in low- and middle-income countries. The objective of this publication is to review how the concept of mental capacity has been defined in Colombian law. We then briefly compare the Colombian and UK situations and propose a few points of discussion, addressing some difficulties and challenges of both countries. Finally, we propose that the first steps in the Colombian context would be to strengthen understanding about mental capacity in medical schools, postgraduate and other health related programmes, the adoption of standardized tools to improve its assessment in everyday clinical settings and the establishment of community care services from collaborative efforts between governmental and civil organizations.

In recent decades, international organisations have created a series of documents proclaiming the protection of the human rights of persons with disabilities (United Nations, 1982, 1991, 2006). The most recent and significant is the United Nations Convention on the Rights of Persons with Disability (CRPD), adapted by the United Nations General Assembly in 2006 (United Nations, 2006). This Convention emphasises in its statements the respect for individual autonomy and their full exercise of rights.

In the UK, clinical communities are starting to become aware of the CRPD and the government is reviewing the compatibility of its laws on mental capacity (Mental Capacity Act, 2005) with its commitments under the CRPD (Martin *et al*, 2016). In Colombia, the government officially adopted the CRPD in 2009 (Ley 1346, 2009). The objective of this publication is to review how the concept of mental capacity has been defined in Colombian law and how the CRPD is likely to impact. We will then briefly compare the Colombian and UK situations and propose a few points of discussion.

## The Colombian case

The notion of legal capacity has been proclaimed in the Colombian legal system since 1887 (Presidencia de la República de Colombia, 1887a; Galindo, 2011), and it is defined in the national Civil Code as the persons' ‘ability or suitability to have rights and obligations’ (Tamayo Lombana, 1997, p. 113). The concept of mental capacity has been implicitly interpreted as equivalent to legal capacity, since the latter presupposes the capacity to exercise these rights by way of one's own conduct and without a third party. Colombian law stipulates that the legal capacity is an ‘attribute or essential quality of the person’ (Tamayo Lombana, 1997), and therefore every individual is considered legally – and mentally – capable except when the law explicitly declares him incapable (Presidencia de la República de Colombia, 1887b). Particularly, the Colombian legislation considers that the person does not have legal (and mental) capacity in two circumstances: when the person is a minor (i.e. less than 18 years old), or when the person presents a mental disorder or medical condition that affects mental capacity (Presidencia de la República de Colombia, 1887c).

In the Colombian context, there are two possible scenarios in which an evaluation of mental capacity could occur: the first is the legal process of interdiction, and the second is when an informed consent is required to proceed with certain medical treatment. In the following sections we will describe in greater detail the most prominent conceptual and practical aspects of both scenarios.

### Interdiction

The Colombian legislation, in law 1306 of 2009 (Ley 1306, 2009), has developed two legal categories with the intention to protect people with mental disabilities, particularly with regard to the management and disposition of their possessions (Presidencia de la República de Colombia, 1970). The first category is the ‘interdiction’, which refers to persons with severe disabilities who have traditionally been regarded as people with an ‘absolute mental incapacity’. Individuals under this legal category are deprived of the power to dispose of their properties, to marry, to handle bank accounts or to contract with public or private entities. They are submitted under the protection of a guardian, who will make all decisions for them. From a legal perspective, they lose all civil rights and there is a grave danger that they cease to be seen as persons. In the second category, ‘relative incapacity’, the mental disability is considered as minor and the law provides a disqualification of specific civil acts or contracts in which such mental disability can hinder the person's decisions.

The process of establishing a person as totally or partially interdicted requires that the interested parties (normally the patient and his family) claim before a family court their wish to start the legal process of interdiction (Presidencia de la República de Colombia, 1979). The judge then asks the Forensic Medicine Institute to conduct a psychiatric evaluation of the relevant person and to report ‘the manifestations of the current state of the patient; the aetiology, diagnosis and prognosis of the disorder, along with its impact on the patient's ability to manage its assets and dispose of them’ (Presidencia de la República de Colombia, 1970). Based on this report, the judge decides whether to declare the person as totally or partially ‘incapable’, or neither.

Interestingly, the Colombian forensic system has no standardised procedures for determining a person's mental capacity. Therefore, forensic psychiatrists depend purely on their clinical judgement and subjective understanding of the concept of mental capacity. Moreover, even in cases where the person with disability has ‘lucid intervals’, or periods of wellness, only decisions accredited by the person's guardian are treated as relevant (Barrera Galvis *et al*, 2012). Furthermore, although the guardian has an ethical duty to the interdicted person, there are many cases of abuse and limited formal oversight.

The legal basis of interdiction therefore involves a total substitution in the process of decision making for people with mental disability, which appears contrary to the ‘right to recognition everywhere as a person before the law’ ratified in article 12 of the CRPD. Thus several international courts have indicated that complete restriction of legal capacity through interdiction constitutes a serious violation of human rights in this population (Fernández, 2010). In Colombia, various governmental and civil society organisations have advocated to repeal or at least substantially change the interdiction process. Part of this effort was codified by law 1618 of 2013 (Ley 1618, 2013).

### Informed consent

Valid consent is an informed, voluntary and capable permission that the patient gives to the doctor for a treatment. Colombia has established the importance of informed consent in medical practice since 1981 through article 15 of law 23 of the same year (Ley 23, 1981). Furthermore, in 1991 the resolution 13437 established the right of the patient to receive information about his condition, procedures, treatments and risks (Ministerio de salud y protección social, 1991). Notably, the Colombian Constitutional Court in verdict T-401 of 1994 (Corte Constitucional, T-401, 1994) and T-216 of 2008 (Corte Constitucional, T-216, 2008) also has mentioned how informed consent is a prerequisite in medical practice to ensure the protection of the constitutional principles of autonomy, justice and beneficence. Finally, in the field of mental health, resolution 2417 of 1992 states that people with mental disorders have ‘the right to be informed about their diagnosis and the most appropriate treatment, and to provide or deny his consent to receive it’ (Ministerio de salud y protección social, 1992). The recent Mental Health Act 1616 of 2013 explicitly states that every person has the right to demand informed consent for any proposed treatment (Ley 1616, 2013).

Despite these legal advances, there are several factors that hinder the application of the informed consent within Colombian mental health services. First, although informed consent is linked to assessment of mental capacity (Plaza & Jiménez, 2015), the Colombian legal framework exclusively stipulates the necessity of this assessment within the process of interdiction (discussed above), with only a minority of cases reaching this formal process. Second, this legal framework does not stipulate any parameters on how to asses mental capacity (either for interdiction or informed consent), and current medicine and psychiatry training programs provide few academic elements on how to perform these assessments. Therefore, most evaluations of mental capacity in clinical settings are based only in the intuitive clinical judgements of the treating physician (Herazo, 2011). Third, informed consent within Colombian psychiatry practice is only used for administrative aspects of electroconvulsive therapy or to proceed with a hospital admission, without implicating a horizontal therapeutic relationship where the patient is involved in decisions about their treatment. In the authors' opinion, the vertical (or paternalistic) doctor–patient relationship is probably explained by a history of poor access to services and low educational background of significant portions of the population. Finally, the transition of the Colombian health system towards an insurance model of health after 2001 has generated a significant reduction in the time for interaction between doctors and patients, limiting the possibility of a therapeutic alliance and effective communication. The short time for evaluation favours a unilateral embrace of therapeutic decisions being made only by the doctor.

## Discussion and conclusions

Several international proclamations have advocated in the past decades for the autonomy of people with mental disorders. Countries such as the UK and the USA have explored the concept of mental capacity integrating systematic assessments in clinical practice (Kim *et al*, 2006; Okai *et al*, 2007; Owen *et al*, 2009, 2013; Szmukler *et al*, 2010). Advances in such countries have been possible given their relatively strong legal systems that are integrated with clinical services, although their health systems and other social institutions have struggled to meet emerging demands during this transition.

Despite Colombia acknowledging the same international guidelines by developing in recent decades a judicial framework that affirms autonomy rights, the country has preserved a more naturalistic practice of psychiatry where clinical concepts (still based on beneficence principles) prevail over legal ones (supporting autonomy), which are actually unfamiliar to most clinicians. It is important to highlight two aspects of this situation in Colombia compared with the UK: patients are not frequently involved in a shared decision-making processes about their treatment because of a weaker culture of autonomy; however, urgently required therapeutic interventions occur more promptly because less time is spent in legal process and the clinical concepts prevail.

Notably, the transition from a substitution model to one of support in decision making involves several challenges for the Colombian state, for courts and for psychiatrists. It is essential to clarify the concepts used by Colombian psychiatrists to evaluate mental capacity, since the absence of national, legal or academic guidelines regarding how to assess it (and how to proceed) results in the full responsibility of the patient being in the hands of the psychiatrist. Therefore, implementing effective and standardised instruments to assess mental capacity in a matter- and time-specific manner should be a priority. For this purpose, several instruments with some international validation – the MacArthur Competence Assessment Tool for Treatment or The Hopkins Competency Assessment Test – could potentially be adopted (Janofsky *et al*, 1992; Appelbaum *et al*, 1997; Dunn *et al*, 2006; Herazo, 2011). In addition, strengthening the academic education about mental capacity in medical schools, postgraduate and other health-related programmes is fundamental.

The Colombian state must also take the necessary measures to provide the support people with disability may require to exercise their rights. Collaborative efforts between governmental and civil organisations to establish community care services sensitive to the human rights perspective could be essential in supporting the most vulnerable people. However, the cost of these services should be carefully weighed, since they need to be adequately resourced to care for the most vulnerable people and to provide a compelling alternative to current models. Legal empowerment of clinicians with clinically and ethically sensitive tools for adequately assessing mental capacity may help transition from the interdiction system, which already places a great burden on governmental institutions, to a system more responsive to the needs and experiences of the most vulnerable people. Finally, it would be valuable to analyse the experiences of the application of decision-making support models in other low- or middle-income countries like Colombia, since it is likely that they have experienced similar difficulties.
